# Impact of Social Drivers of Health, Self-Efficacy, and Substance Use on COVID-19 Preventative Behaviors Among Persons Who Inject Drugs with Hepatitis C: The HERO Study

**DOI:** 10.3390/ijerph23010093

**Published:** 2026-01-09

**Authors:** Snehal S. Lopes, Irene Pericot-Valverde, Paula J. Lum, Lynn E. Taylor, Shruti H. Mehta, Judith I. Tsui, Judith Feinberg, Arthur Y. Kim, Brianna L. Norton, Kimberly Page, Cristina Murray-Krezan, Jessica Anderson, Alison Karasz, Julia Arnsten, Phillip Moschella, Moonseong Heo, Alain H. Litwin, the HERO Study Group

**Affiliations:** 1 Department of Public Health Sciences, Clemson University, Clemson, SC 29634, USA; snehall@clemson.edu (S.S.L.);; 2 Department of Psychology, College of Behavioral, Social, and Health Sciences, Clemson University, Clemson, SC 29634, USA; 3 Department of Medicine, University of California San Francisco, 1001 Potrero Ave, San Francisco, CA 94110, USA; 4 Department of Pharmacy Practice and Clinical Research, University of Rhode Island, 7 Greenhouse Road, Kingston, RI 02881, USA; letaylor@uri.edu; 5 Department of Epidemiology, Johns Hopkins Bloomberg School of Public Health, 615 N. Wolfe Street, Room E6546, Baltimore, MD 21205, USA; 6 Department of Medicine, University of Washington, 325 9th Ave., Seattle, WA 98104, USA; 7 Department of Behavioral Medicine and Psychiatry, West Virginia University School of Medicine, 930 Chestnut Ridge Road, Morgantown, WV 26505, USA; 8 Division of Infectious Diseases, Department of Medicine, West Virginia University School of Medicine, 1 Medical Center Drive, Morgantown, WV 26506, USA; 9 Division of Infectious Diseases, Massachusetts General Hospital, 55 Fruit St., Boston, MA 02114, USA; 10 Harvard Medical School, Harvard University, Boston, MA 02115, USA; 11 Department of Medicine, Albert Einstein College of Medicine, Bronx, NY 10461, USA; 12 Department of Medicine, Montefiore Medical Center, Bronx, NY 10467, USA; 13 Department of Internal Medicine MSC 10 5550, University of New Mexico Health Sciences Center, University of New Mexico, Albuquerque, NM 87131, USA; 14 Department of Medicine, University of Pittsburgh, Pittsburgh, PA 15215, USA; 15 UMass Chan Medical School, University of Massachusetts Medical School, 55 Lake Ave, North Worcester, MA 01605, USA; 16 Department of Emergency Medicine, Prisma Health, Greenville, SC 29605, USA; 17 School of Health Research, Clemson University, Clemson, SC 29634, USA; 18 Department of Medicine, University of South Carolina School of Medicine, 876 W Faris Rd, Greenville, SC 29605, USA; 19 Department of Medicine, Prisma Health, Greenville, SC 29605, USA

**Keywords:** SDoH, self-efficacy, COVID-19 personal protective behaviors, PWID, HCV

## Abstract

Background: Personal protective measures help prevent infection and disease transmission during health crises such as Coronavirus disease 2019 (COVID-19). Populations facing barriers to adhering to these measures are more vulnerable to the health crisis. This study investigated the association of social drivers of health (SDoH), self-efficacy, and adverse substance use behavior changes with ability to practice COVID-19 personal protective behaviors among persons who inject drugs (PWID) with hepatitis C virus (HCV) infection history. Methods: This study used the Hepatitis C Real Options (HERO) study’s COVID-19 survey data (*n* = 157). The association of inability to practice COVID-19 personal protective behaviors (hand washing, social distancing, etc.) with (a) SDoH difficulties (employment, housing, etc.); (b) adverse substance use behavior change (overdose, injecting behavior, etc.); and (c) self-efficacy was tested using logistic regression. Results: Inability to practice any personal protective behaviors was more likely among those experiencing any vs. no SDoH difficulties [adjusted odds ratio (*aOR*) (95% confidence interval (*CI*))] = 4.57 (1.57, 16.40); *p* = 0.003] but less likely for those with higher overall self-efficacy [*aOR* (95% *CI*) = 0.55 (0.32, 0.93); *p* = 0.025] and self-efficacy for setting goals [*aOR* (95% *CI*) = 0.63 (0.40, 0.96); *p* = 0.031]. The association between adverse substance use behavior changes and the outcome was not significant. Conclusions: Greater SDoH difficulties and lower self-efficacy were associated with greater inability to practice COVID-19 personal protective behaviors. Interventions to meet SDoH-related challenges and increase self-efficacy could help encourage practice of personal protective behaviors and economically reduce disease burden during health crises.

## 1. Introduction

During emerging health crises such as the coronavirus disease of 2019 (COVID-19) pandemic, when there was a shortage of personal protective equipment [1] and pharmaceutical measures for prevention/cure were still unknown, non-pharmaceutical public health interventions such as isolation, quarantine, social distancing, and hygiene, became an important first line of defense that individuals in the population have [2,3]. The practice of these personal protective behaviors for managing the spread of communicable diseases in the population has been shown to be effective and was recommended during the COVID-19 pandemic [2,4].

However, promoting adherence to the recommended personal protective behaviors during the COVID-19 pandemic was a challenge [5]. Several factors, including government policies, economic burden of maintaining adherence, social drivers of health (SDoH) (Note: There have been changes in terminology concerning SDoH where by the term ‘social determinants’ is being replaced by ‘social drivers’ to reflect that the social factors may be interpreted as being modifiable [6,7]), risk perceptions, psychological fatigue, health-related self-efficacy, trust in institutions (e.g., government), may have influenced the uptake of personal protective behaviors during the COVID-19 pandemic [5,8]. Among individuals with substance use disorders [which includes persons who inject drugs (PWID) and persons with other comorbidities such as hepatitis C virus (HCV) infection and human immunodeficiency virus (HIV) infection], substance use behaviors may have influenced their level of adherence to the personal protective behaviors [9].

It is important to investigate the factors relating to the practice of personal protective behaviors and address them to prevent future health emergencies from escalating to a global pandemic level. The practice of COVID-19 personal preventative behaviors was especially important for people with substance use disorders for several reasons. The behavioral factors associated with substance use, such as acquiring and sharing of drug and drug injection equipment, in-person meetings, physical closeness or contact, and use of unsanitary equipment, increased the risk of infection [10,11]. Moreover, people with substance use disorders have comparatively poorer health status due to increased physical co-morbidities [including conditions such as HCV and HIV], compromised immune systems, and greater SDoH-related difficulties, all of which make them more susceptible to infectious diseases and increase their risk of disease severity and mortality [12,13].

Even after pharmaceutical measures for prevention/cure become available, access to these measures could still be harder for the substance user population [14]. Also, most research on disease prevention, cure, and management of diseases in the general population fails to include the specific risk factors faced by people with substance use disorders, thus increasing their risk of treatment complications [15].

Despite the importance of promoting personal protective behaviors among people with substance use disorders during a health crisis, few studies have explored factors influencing the COVID-19 personal protective behaviors among people with substance use disorders, and fewer have focused on PWID or PWID with HCV. PWID with HCV infection may be a group more vulnerable to the adverse impact of the COVID-19 pandemic due to the added burden of comorbidity [12,13] and risky injection use behaviors [10,11].

The Hepatitis C Real Options (HERO) study [16,17] was a randomized controlled clinical trial among PWID with HCV infection across multiple sites spread across eight states in the United States (US). The HERO study primarily aimed to investigate the effect of two models of HCV treatment [Patient Navigation (PN) and modified Directly Observed Treatment (mDOT)] on rates of treatment initiation, adherence, and completion; and achievement of HCV cure. The COVID-19 pandemic emerged when the HERO study was in the follow-up phase. In response to the urgent need for COVID-19-related outcomes data during the emerging pandemic, a COVID-19 pandemic-related survey was added to the HERO study.

Using data from the HERO study and the COVID-19 pandemic-related survey introduced into the HERO study [16,17], the current study was conducted with the aim of testing the association of environmental factors (e.g., SDoH difficulties), psychological factors (e.g., health self-efficacy), and behavioral factors (e.g., changes in substance use behaviors) with the ability to practice COVID-19-related personal protective behaviors. The specific objectives were as follows to test the association of ability to practice recommended personal protective behaviors for COVID-19 with (a) SDoH difficulties, including difficulties with employment, childcare, health insurance, housing, finances, and paying for other basic needs; (b) health self-efficacy (overall and specific domains, namely, setting and meeting goals, being in control, having a positive effect on, and working to improve one’s health); and (c) adverse substance use behaviors (including having an overdose, alcohol consumption, drug use, injecting behavior, and needle sharing). The SDoH factors analyzed in this study align with the framework of SDoH domains provided as part of the Healthy People 2030 initiative’s priority areas [18] as follows: employment, sources financial support, and paying for other basic needs relate to the ‘Economic Stability’ domain, the health insurance factor relates to the ‘Health Care Access and Quality’ domain, the childcare factor relates to the ‘Social and Community Context’ and ‘Economic Stability’ domains, and the housing factor relates to the ‘Neighborhood and Built Environment’ and ‘Economic Stability’ domains.

## 2. Materials and Methods

### 2.1. Study Design, Data, and Population

This was a secondary analysis study with an observational design. The sample for this study (*n* = 157) was a subset of participants from the HERO study [16,17] who responded to a COVID-19 pandemic-related survey conducted at a single timepoint between 1 August 2020, and 31 December 2020, while the HERO study was in the follow-up phase. The sample that responded to the HERO study’s COVID-19 pandemic-related survey was 20% of the parent trial’s intention-to-treat sample, and 25% of the sample that initiated treatment as part of the HERO study. No specific or additional exclusion or inclusion criteria were applied to this survey, beyond those of the parent HERO study. Thus, this sample is a convenience sample for this particular HERO contingent study. All the participants were PWID who had HCV infection at the time of enrollment into the HERO study, but most of the study sample [*n* = 139 (88.5%)] had achieved sustained virologic response (SVR, equivalent to HCV cure) before the time the COVID-19 pandemic-related survey was conducted. The data used in this study included that collected as part of the main HERO study, as well as that collected as part of the HERO study’s COVID-19 pandemic-related survey. The HERO study was conducted in accordance with the Declaration of Helsinki and approved by the Institutional Review Board at each of the participating institutions. Informed consent was obtained from all subjects involved in the study.

### 2.2. Independent Variables

#### 2.2.1. SDoH Difficulties

The HERO study’s COVID-19 pandemic-related survey asked participants whether they experienced any difficulties with specific SDoH factors due to the COVID-19 pandemic, including employment, sources of financial support, housing, childcare, health insurance, and paying for other basic needs (see Appendix A for the items used in the instrument). Each of the specific SDoH factors measured in this study was analyzed as a separate independent variable. An additional variable (“any SDoH loss”) indicating whether participants reported difficulty with any SDoH factor (including employment, sources of financial support, housing, childcare, health insurance, and other basic needs) was also analyzed as an independent variable.

#### 2.2.2. Health Self-Efficacy

Health self-efficacy was measured as part of the main HERO study using a five-item scale [19] where higher scores implied a higher level of self-efficacy. Self-efficacy was measured at baseline (week 0) and at the end of HCV direct-acting antiviral treatment (week 12). For this study, we used the self-efficacy data from week 12, because it was closer to the COVID-19 survey. If week 12 self-efficacy data were missing, baseline values were used (for *n* = 13 participants). The overall self-efficacy scores as well as the individual item scores were analyzed as independent variables in this study.

#### 2.2.3. Adverse Changes in Substance Use Behaviors Ensuing from the COVID-19 Pandemic

Participants were asked if they had experienced an overdose due to the COVID-19 pandemic (‘yes’/‘no’). Additional questions measured changes in alcohol consumption, drug use, injecting behavior, and needle sharing behaviors using the response options ‘More than usual’, ‘Less than usual’, ‘Nothing changed’, ‘No’ and ‘Not applicable’. For this study, these responses were collapsed into a binary variable coded as ‘More than usual’ = 1, and the rest of the responses = 0. A composite binary variable indicating adverse change in any substance use behavior (including overdose, alcohol consumption, drug use, injecting behavior, and needle sharing) was also analyzed as an independent variable.

### 2.3. Outcome: Inability to Practice Any COVID-19 Personal Protective Behaviors

The HERO study’s COVID-19 survey measured the practice of the following five recommended personal protective behaviors for COVID-19: hand washing/sanitizing, disinfecting surfaces, social distancing, home quarantining/self-isolating, and avoiding large group gatherings. Participants provided information about their practice of each of these personal protective behaviors (see Appendix B for the items used in the instrument) using the response options (a) ‘yes’, (b) ‘no, I don’t see the need to do this’, and (c) ‘no, I am unable to do this’. Inability to practice any COVID-19 personal protective behaviors was a binary variable coded as ‘1 = unable’ if the participant selected the ‘no, I am unable to do this’ response option for any of the five COVID-19 personal protective behaviors measured in this study and coded as ‘0 = able’ otherwise.

### 2.4. Statistical Analyses

Baseline sociodemographic and clinical characteristics of the study population were explored using descriptive statistics and were compared between the two levels of the outcome using chi-square tests (or Fisher’s exact tests if expected frequencies for more than 20% of the cells were <5) for categorical characteristics and independent samples *t*-tests for continuous variables (or Wilcoxon rank sum tests for continuous variables with a non-normal distribution). Associations between each independent variable and the outcome were explored using exact logistic regression. Each of the regression models adjusted for the baseline characteristics that were significantly different between the outcome levels: self-report of having injected heroin, and a mixture of cocaine and heroin in the three months prior to baseline. The association of the outcome with self-efficacy was also tested in the sample (*n* = 118) that excluded those who indicated ‘no, I don’t see the need to do this’ for all the measured COVID-19 personal protective behaviors. All analyses were conducted using SAS software, version 9.4 (SAS Institute Inc., Cary, NC, USA). The analyses were conducted in 2025.

## 3. Results

### 3.1. Baseline Characteristics of the Study Sample

The study sample was 43.3 (SD = 11.1) years of age on average and included a majority of males (*n* = 103; 65.6%), persons from White/Caucasian race (*n* = 105; 67.7%), and non-Hispanic ethnicity (*n* = 132; 84.1%). Most reported their relationship status as being single, separated, divorced, or widowed (*n* = 143; 91.1%). The highest achieved educational level was high school or less for most participants (*n* = 92; 58.6%), and a large proportion were unemployed (*n* = 98; 62.4%), had unstable housing (*n* = 85; 54.1%), and did not have their own transportation (*n* = 83; 52.9%). Comparison of baseline characteristics between those able and unable to practice COVID-19 personal protective behaviors indicated significant differences with respect to self-report of injecting heroin and mixture of cocaine and heroin. Participants self-reporting having injected heroin were significantly more likely to report being unable to practice any of the COVID-19 personal protective compared to those who did not self-report injecting heroin (30.2% versus 12.1%, *p* = 0.037). Similarly, participants self-reporting having injected a mixture of cocaine and heroin were significantly more likely to report being unable to practice any of the COVID-19 personal protective measures compared to those who did not self-report injecting the mixture (38.9% versus 22.1%, *p* = 0.046). Additional characteristics and comparisons of the characteristics between those who were able and unable to practice COVID-19 personal protective behaviors are presented in Table 1.

### 3.2. SDOH Factors and Practice of COVID-19 Personal Protective Behaviors

In the sample study, 42 participants (26.8%) reported an inability to practice any of the personal protective behaviors. Table 2 presents the results of analyses examining the association between SDoH factors and the practice of COVID-19 personal protective behaviors. Inability to practice any of the COVID-19 personal protective behaviors was more likely among participants experiencing any SDoH difficulty compared to those not experiencing any SDoH difficulty [*aOR* (95% *CI*) = 4.57 (1.57, 16.40); *p* = 0.003], among those experiencing childcare difficulties compared to those not experiencing childcare difficulties [*aOR* (95% *CI*) = 3.39 (1.06, 10.95); *p* = 0.040], among those affected by housing loss compared to those not experiencing housing loss [*aOR* (95% *CI*) = 6.05 (1.43, 30.81); *p* = 0.012], and among those with difficulties paying for basic needs compared to those without difficulties paying for basic needs [*aOR* (95% *CI*) = 2.34 (1.01, 5.51); *p* = 0.047].

### 3.3. Health Self-Efficacy and Practice of COVID-19 Personal Protective Behaviors

Table 3 presents the results of analyses examining the association between health self-efficacy and the ability to practice any of the COVID-19 personal protective behaviors. The likelihood of being unable to practice any of the COVID-19 personal protective behaviors was lower for each unit increase in overall self-efficacy [*aOR* (95% *CI*) = 0.55 (0.32, 0.93); *p* = 0.025], and self-efficacy for setting goals [*aOR* (95% *CI*) = 0.63 (0.40, 0.96); *p* = 0.031]. The other domains of self-efficacy were not significantly associated with the ability to practice the COVID-19 personal protective behaviors. The subgroup (*n* = 118) that excluded those who did not believe in practicing any of the COVID-19 personal protective behaviors had 34 (28.81%) participants reporting inability to practice any of the COVID-19 personal protective behaviors. The findings for this subgroup showed that the effects were similar to those of the total sample, but none of the associations reached statistical significance.

### 3.4. Adverse Changes in Substance Use and Practice of COVID-19 Personal Protective Behaviors

Table 4 shows the results of analyses testing the association of adverse changes in substance use with the practice of COVID-19 personal protective behaviors. The likelihood of being unable to practice any of the COVID-19 personal protective behaviors did not differ significantly by experience of adverse changes in the different substance use behaviors. However, the direction of effects suggested that there was a greater likelihood of being unable to practice COVID-19 personal protective behaviors for those who were classified as having an adverse change in the drug use-related behaviors compared to those who were not classified as having the same behavior. For instance, those experiencing an increase in injection drug use had a higher likelihood (though not significantly) of being unable to practice the COVID-19 personal protective behaviors compared to those not experiencing an increase in injection drug use [*aOR* (95% *CI*) = 1.93 (0.61, 5.81); *p* = 0.296].

## 4. Discussion

This study, based on a sample of PWID with HCV infection or a history of HCV infection, aimed to understand how SDoH, health self-efficacy, and adverse changes in substance use related to the practice of recommended COVID-19 personal protective behaviors. The key findings of this study are (a) experiencing adverse changes in SDoH factors due to COVID-19, especially with respect to difficulties with childcare, housing loss, and paying for basic necessities were significantly associated with reduced ability to practice COVID-19 personal protective behaviors; (b) higher overall self-efficacy, and self-efficacy for setting goals were significantly associated with improved ability to practice the COVID-19 personal protective behaviors; and (c) the association of adverse changes in substance use and the ability to practice COVID-19 personal protective behaviors was not significant.

Although personal protective measures are beneficial for limiting the spread of infection, they also have social and health costs [20,21]. For instance, school closures meant an additional burden of providing meals and childcare alongside job responsibilities [20]. Social distancing measures were more burdensome for those with greater healthcare needs but lesser or no access to telemedicine, and a lack of ability to use telemedicine [21,22]. Adherence to social distancing was not feasible, if not near impossible, for those living in overcrowded settings, multigenerational households [23], and in settings such as prisons [24]. Practicing personal hygiene requires access to amenities (clean water, soap/sanitizer, space for washing), which were accessible to persons facing housing difficulties or those struggling to meet basic needs [25,26]. Prior studies in other populations (e.g., persons with cardiovascular disease, older adults) have also found that a greater burden of SDoH difficulties is associated with a lower likelihood of practicing COVID-19 personal protective behaviors [27,28]. Among people with substance use disorders, a prior study in New York City showed that street homelessness was associated with reduced ability to practice personal protective behaviors [29]. Therefore, although personal protective behaviors appear technically simplistic, there are a host of SDoH-related barriers that must be addressed to enable people to adhere to the recommended behaviors. For people from challenging SDoH backgrounds, practice of every recommended personal protective behavior may not be feasible. But interventions could aim to spread awareness about the various options to protect oneself from infection and encourage the practice of feasible options. For example, always wearing a mask and avoiding contact through touch should be emphasized if avoiding crowded settings is not possible. Providing easier access to protective equipment (masks, hygiene products) would help improve adherence to some personal protective behaviors. Alternate strategies for self-protection besides the standard best practices recommendations need to be developed, considering the infrastructure, amenities, lifestyles, and culture within lower socio-economic contexts, similar to how special personal protective practices were developed for the healthcare context where workers had to be on the frontlines [30].

Our study is among the first to investigate whether self-efficacy may relate to practice of COVID-19 personal protective measures among PWID with recent HCV infection—a population that has a high burden of multiple comorbidities but experiences manifold barriers to adhering to required health protective behaviors [31,32,33]. Higher health self-efficacy was related to a lower likelihood of being unable to practice any of the COVID-19-related personal protective behaviors, which could be because self-motivation and the confidence to act have a more important role to play in contexts ridden by manifold barriers to the recommended behaviors. Self-efficacy, a motivational factor, has been associated with the practice of COVID-19 preventative behaviors in other populations too [8,34,35]. With respect to the self-efficacy domains, our findings showed that among PWID, higher self-efficacy related to setting goals was associated with a lower likelihood of being unable to practice any of the COVID-19 personal protective behaviors. This could be because setting goals relates to the ability to self-regulate one’s behavior [36]. Persons with better self-regulation skills may have been more capable of achieving the behavior change required for personal protection during the COVID-19 pandemic. Therefore, interventions aiming to promote the practice of recommended health behaviors among PWID could include psychological support to raise levels of health self-efficacy.

The association of COVID-19 personal protective behaviors with adverse changes in substance use was not significant in this study. However, the direction of effects for all the drug use-related outcomes (any drug use, injecting, needle sharing, overdosing) suggested a higher likelihood of being unable to practice the behaviors among those experiencing adverse changes for the drug use outcomes, compared to those not experiencing adverse changes for the drug use outcomes due to the pandemic. This potential link between adverse substance use and COVID-19 personal protective behaviors aligns with findings of prior studies in the general adult population without substance use disorders, which suggested that drug use was associated with lower compliance with social distancing [9,37]. During the COVID-19 pandemic period, the operations of substance use treatment and harm reduction services were affected [15,38]. People with substance use disorders often had to go to additional lengths to acquire drugs, involving sharing activities and use of unhygienic equipment [10,11], which meant that adherence to COVID-19 personal protective behaviors may have been compromised. Addressing substance use disorders may be a critical pathway to promoting adherence to personal protective behaviors recommended during public health crises.

The limitations of this study are as follows: (a) As the COVID-19 pandemic and pandemic-related policies evolved, the COVID-19 personal protective behaviors [5] and SDoH difficulties [39] also changed over time, but the data from this study were measured at a single time point. (b) The last available measure of self-efficacy collected in the pre-COVID-19 period was used. Self-efficacy levels during the pandemic period may be different. However, it is useful to analyze the association of self-efficacy levels prior to the pandemic with personal protective behaviors during the pandemic because interventions to improve health-related self-efficacy levels during non-crisis times could enhance preparedness for dealing with future health crises and save valuable time and resources when the crisis emerges. (c) Since the data used in this study were collected during the COVID-19 pandemic, and it has been about five years since the pandemic ended, the timeliness of the findings from this study may be viewed as limited from the public health perspective. However, COVID-19 is still the latest pandemic, and thus at present, it is the best reference point for guiding health preparedness efforts in terms of health awareness and education to mitigate impacts of a future potential pandemic, which may involve either novel respiratory viruses or pandemic influenza. (d) Health self-efficacy may not correlate with self-efficacy specific to COVID-19 personal protective behaviors. (e) The sample included in this study was a convenience sample. The results may differ for participants who were unreachable or did not respond to the HERO study’s COVID-19 pandemic survey. (f) Due to small sample sizes, this study may not be sufficiently powered to detect the effects. (g) The study was able to analyze only those SDoH factors, self-efficacy domains, and recommended COVID-19 personal protective behaviors that were measured in this study. For example, mask-wearing behavior, which was highly recommended as a form of personal protection, was not included. (h) This is an exploratory study, where the relationships found should not be interpreted as causal.

## 5. Conclusions

Inability to practice personal protective behaviors for COVID-19 among PWID with HCV or a history of HCV infection was associated with having experienced SDoH difficulties brought in by the pandemic and with lower levels of health self-efficacy. In the PWID population with a history of HCV infection, being unable to practice recommended COVID-19 personal protective behaviors was associated with increased likelihood of COVID-19 infection. COVID-19 infection among PWID with HCV implies an added burden of managing co-infections and may lead to more adverse COVID-19 outcomes. Therefore, providing support to address SDoH-related challenges and implementing interventions to increase health-related self-efficacy are important for promoting the adoption of personal protective behaviors and help economically reduce the burden of disease during health crises such as the COVID-19 pandemic.

## Figures and Tables

**Table 1:** Baseline characteristics of the study sample.

Characteristics		Ability to Practice COVID-19 Personal Protective Behaviors:		
	Total Study Sample (*n*= 157)	Able (*n* = 115)	Unable (*n* = 42)	*p*
Treatment Arm				0.222
*mDOT*	66	45 (68.2%)	21 (31.8%)	
*PN*	91	70 (76.9%)	21 (23.1%)	
Gender				0.426
*Female*	52	35 (67.3%)	17 (32.7%)	
*Male*	103	78 (75.7%)	25 (24.3%)	
*Transgender or Gender* *Non-conforming*	2	2 (100%)	0 (0.0%)	
Age [Mean (SD)]	43.3 (11.1)	44.3 (10.9)	40.6 (11.4)	0.065
Race				0.535
*White/Caucasian*	105	75 (71.4%)	30 (28.6%)	
*Black/African American*	27	22 (81.5%)	5 (18.5%)	
*Other*	23	16 (69.6%)	7 (30.4%)	
*Not Available*	2	2	0	
Latino/Hispanic Ethnicity				0.735
*No*	132	96 (72.7%)	36 (27.3%)	
*Yes*	25	19 (76.0%)	6 (24.0%)	
Marital/cohabitation Status				0.288
*Single/Separated* */Divorced/Widowed*	143	102 (71.3%)	41 (28.7%)	
*Married/living together* *as married*	12	11 (91.7%)	1 (8.3%)	
*Other*	2	2 (100%)	0 (0.0%)	
Education				0.684
*Less than HS*	36	28 (77.8%)	8 (22.2%)	
*HS diploma or GED*	56	39 (69.6%)	17 (30.4%)	
*Some college or more*	65	48 (73.8%)	17 (26.2%)	
Living stability				0.413
*Stable housing*	72	55 (76.4%)	17 (23.6%)	
*Unstable housing*	85	60 (70.6%)	25 (29.4%)	
Availability of transportation				0.333
*Yes*	74	57 (77.0%)	17 (23.0%)	
*Maybe, if I can get a* *ride*	9	7 (77.8%)	2 (22.2%)	
*Maybe, if public* *transportation is* *available*	71	50 (70.4%)	21 (29.6%)	
*No*	3	1 (33.3%)	2 (66.7%)	
Employed with a regular job or informal work				0.771
*Yes*	59	44 (74.6%)	15 (25.4%)	
*No*	98	71 (72.4%)	27 (27.6%)	
Clinical Setting Location				0.057
*Boston*	24	18 (75.0%)	6 (25.0%)	
*Providence*	49	32 (65.3%)	17 (34.7%)	
*New York City*	2	2 (100%)	0 (0.0%)	
*Baltimore*	29	20 (69.0%)	9 (31.0%)	
*Morgantown*				
*Albuquerque*	13	13 (100%)	0 (0.0%)	
*San Francisco*	31	21 (67.7%)	10 (32.3%)	
*Seattle*	9	9 (100%)	0 (0.0%)	
Clinical Setting Type				0.982
*OTP*	82	60 (73.2%)	22 (26.8%)	
*CHC*	75	55 (73.3%)	20 (26.7%)	
Medication for OUD				0.664
*None*	32	22 (68.8%)	10 (31.3%)	
*Buprenorphine only*	12	8 (66.7%)	4 (33.3%)	
*Any Methadone*	113	85 (75.2%)	28 (24.8%)	
Depressive symptoms (PHQ-9) [Mean (SD)]	9.9 (6.5)	10.1 (6.7)	9.5 (5.7)	0.965
Anxiety symptoms (GAD-7) [Mean (SD)]	8.5 (6.0)	8.4 (6.0)	8.8 (6.1)	0.743
*Not Available*	1	1	0	
HIV infection (positive)				0.605
*No*	86	66 (76.7%)	20 (23.3%)	
*Yes*	27	22 (81.5%)	5 (18.5%)	
*Not Available*	44	27	17	
Alcohol misuse				0.551
*No*	116	86 (74.1%)	30 (25.9%)	
*Yes*	39	27 (69.2%)	12 (30.8%)	
*Not Available*	2	2	0	
Last drug injection (within 3 months of screening)				0.536
*0–4 weeks*	121	87 (71.9%)	34 (28.1%)	
*5–8 weeks*	23	19 (82.6%)	4 (17.4%)	
*9–12 weeks*	13	9 (69.2%)	4 (30.8%)	
Number of days injected drugs in the past 3 months [Mean (SD)]	33.3 (30.8)	32.1 (29.4)	36.7 (34.8)	0.755
*Not Available*	1	0	1	
Times injecting drugs a day [Mean (SD)]	3.0 (3.2)	3.0 (3.5)	3.0 (1.9)	0.155
Drug injection reported within 3 months of baseline				0.442
*No*	8 (100%)	5 (62.5%)	3 (37.5%)	
*Yes*	149 (100%)	110 (73.8%)	39 (26.2%)	
Substances injected in the past 3 months among those reporting injecting (*n* = 149):				
Mixture of cocaine and heroin				**0.046**
*No*	113	88 (77.9%)	25 (22.1%)	
*Yes*	36	22 (61.1%)	14 (38.9%)	
Mixture of methamphetamine and heroin				0.557
*No*	123	92 (74.8%)	31 (25.2%)	
*Yes*	26	18 (69.2%)	8 (30.8%)	
Heroin				**0.037**
*No*	33	29 (87.9%)	4 (12.1%)	
*Yes*	116	81 (69.8%)	35 (30.2%)	
Methamphetamine				0.844
*No*	105	78 (74.3%)	27 (25.7%)	
*Yes*	44	32 (72.7%)	12 (27.3%)	
Cocaine				0.310
*No*	105	80 (76.2%)	25 (23.8%)	
*Yes*	44	30 (68.2%)	14 (31.8%)	
Crack				0.168
*No*	125	95 (76.0%)	30 (24.0%)	
*Yes*	24	15 (62.5%)	9 (37.5%)	
Fentanyl				0.642
*No*	8	6 (75.0%)	2 (25.0%)	
*Yes*	12	7 (58.3%)	5 (41.7%)	
*Not Available*	137	102	35	
Poly-substances				0.109
*No*	66	53 (80.3%)	13 (19.7%)	
*Yes*	83	57 (68.7%)	26 (31.3%)	
*Not Available*	8	5	3	
Urine drug screen conducted at baseline visit				0.191
*No*	7	7 (100%)	0 (0.0%)	
*Yes*	150	108 (72.0%)	42 (28.0%)	
Urine drug screen results at baseline visit among those screened (*n* = 150):				
Any drug				0.673
*No*	6	4 (66.7%)	2 (33.3%)	
*Yes*	144	104 (72.2%)	40 (27.8%)	
Amphetamine				0.622
*Positive*	40	30 (75.0%)	10 (25.0%)	
*Negative*	110	78 (70.9%)	32 (29.1%)	
Methamphetamine				0.579
*Positive*	45	31 (68.9%)	14 (31.1%)	
*Negative*	105	77 (73.3%)	28 (26.7%)	
Benzodiazepine				0.383
*Positive*	88	61 (69.3%)	27 (30.7%)	
*Negative*	62	47 (75.8%)	15 (24.2%)	
Cocaine				0.280
*Positive*	61	41 (67.2%)	20 (32.8%)	
*Negative*	89	67 (75.3%)	22 (24.7%)	
THC/Cannabis				0.988
*Positive*	68	49 (72.1%)	19 (27.9%)	
*Negative*	82	59 (72.0%)	23 (28.0%)	
Opiate				0.256
*Positive*	71	48 (67.6%)	23 (32.4%)	
*Negative*	79	60 (75.9%)	19 (24.1%)	
Oxycodone				0.229
*Positive*	35	28 (80.0%)	7 (20.0%)	
*Negative*	115	80 (69.6%)	35 (30.4%)	

Note: Abbreviations [mDOT (modified Directly Observed Treatment), PN (Patient Navigation), PHQ-9 (9-item Patient Health Questionnaire [17]), GAD-7 (7-item General Anxiety Disorder [18]), THC (tetrahydrocannabinol), OTP (Opioid Treatment Program), CHC (Community Health Center)]; *p*-values significant at the 5% threshold are in bold font.

**Table 2:** Association of SDoH difficulties with inability to practice COVID-19 personal protective behaviors.

SDOH Difficulties	Practice of Any COVID-19 Personal Protective Behaviors					
	Able (*n* = 115)		Unable (*n* = 42)			
	*n*	%	*n*	%	*aOR* * (95% *CI*)	*p*
Any SDOH loss ^1^						
*No*	44	88.00	6	12.00	4.57 (1.57, 16.40)	**0.003**
*Yes*	70	66.04	36	33.96		
Job Loss/reduced work hours						
*No*	68	75.56	22	24.44	1.28 (0.56, 2.93)	0.644
*Yes*	46	69.70	20	30.30		
Childcare difficulties						
*No*	104	76.47	32	23.53	3.39 (1.06, 10.95)	**0.040**
*Yes*	10	50.00	10	50.00		
Loss of other sources of financial support						
*No*	89	71.77	35	28.23	0.70 (0.21, 2.04)	0.659
*Yes*	25	78.13	7	21.88		
Loss of housing/becoming homeless						
*No*	109	76.76	33	23.24	6.05 (1.43, 30.81)	**0.012**
*Yes*	5	35.71	9	64.29		
Loss of health insurance						
*No*	111	73.03	41	26.97	1.43 (0.02, 33.56)	1.000
*Yes*	2	66.67	1	33.33		
Difficulties paying for basic needs						
*No*	73	78.49	20	21.51	2.34 (1.01, 5.51)	**0.047**
*Yes*	38	63.33	22	36.67		
*Not Available*	1		0		(not included in the regression models)	

Note: * Adjusted Odds Ratio (*aOR*) indicating the likelihood of being unable to practice COVID-19 personal protective behaviors for the ‘yes’ vs. the ‘no’ (reference group) categories for each of the SDOH difficulty types; ^1^ ‘Any SDoH loss’ is a binary variable indicating whether participants reported difficulty with any of the SDoH factors (job loss, childcare, other sources of financial support, housing, health insurance, and other basic needs); *p*-values significant at the 5% threshold are in bold font.

**Table 3:** Association of self-efficacy with practice of COVID-19 personal protective behaviors.

Health Self-Efficacy	M	SD	M	SD	*aOR* ^1^ (95% *CI*)	*p*
	Practice of any COVID-19 protective behaviors					
[Total sample (*n* = 157)]	Able (*n* = 115)		Unable (*n* = 42)			
Overall health self-efficacy score	4.07	0.72	3.80	0.70	0.55 (0.32, 0.93)	**0.025**
*Health self-efficacy domains:*						
a. Setting goals to improve one’s health	4.17	0.87	3.83	0.91	0.63 (0.40, 0.96)	**0.031**
b. Meeting goals to improve one’s health	3.65	0.95	3.38	1.08	0.68 (0.44, 1.02)	0.065
c. Having a positive effect on one’s health	4.19	0.88	4.00	0.88	0.73 (0.47, 1.14)	0.174
d. Being in control of learning about one’s health	4.17	0.91	3.90	0.85	0.72 (0.47, 1.11)	0.140
e. Working to improve one’s health	4.17	0.80	3.90	0.88	0.65 (0.40, 1.04)	0.074
[Subgroup ^2^ (*n* = 118)]	Able (*n* = 84)		Unable (*n* = 34)			
Overall health self-efficacy score	4.09	0.71	3.79	0.64	0.54 (0.28, 1.01)	0.055
*Health self-efficacy domains:*						
a. Setting goals to improve one’s health	4.20	0.80	3.85	0.82	0.62 (0.35, 1.06)	0.084
b. Meeting goals to improve one’s health	3.67	0.94	3.35	0.98	0.69 (0.42, 1.12)	0.146
c. Having a positive effect on one’s health	4.21	0.88	3.97	0.87	0.69 (0.41, 1.18)	0.189
d. Being in control of learning about one’s health	4.20	0.85	3.88	0.88	0.65 (0.38, 1.08)	0.099
e. Working to improve one’s health	4.15	0.81	3.91	0.75	0.72 (0.41, 1.25)	0.266

Note: *p*-values significant at the 5% threshold are in bold font. ^1^ Adjusted Odds Ratio (*aOR*) indicating the likelihood of being unable to practice COVID-19 personal protective behaviors for each unit increase in the self-efficacy domain; ^2^ Results of analyses within the subgroup (*n* = 118) that excluded those who did not believe in practicing all of the COVID-19 personal protective behaviors.

**Table 4:** Association of adverse changes in substance use behaviors with inability to practice COVID-19 personal protective behaviors.

Substance Use Behavior Change Due to COVID-19 Pandemic	Practice of Any COVID-19 Personal Protective Behaviors					
	Able (*n* = 115)		Unable (*n* = 42)			
	*n*	%	*n*	%	*aOR* * (95% *CI*)	*p*
Adverse change in overall substance use behavior						
*No*	84	76.36	26	23.64	1.75 (0.72, 4.23)	0.244
*Yes*	31	65.96	16	34.04		
Increased alcohol use						
*No*	103	73.57	37	26.43	0.90 (0.15, 3.93)	1.000
*Yes*	12	70.59	5	29.41		
Increased drug use						
*No*	97	75.78	31	24.22	1.98 (0.70, 5.42)	0.217
*Yes*	18	62.07	11	37.93		
Increased injection drugs use						
*No*	101	75.37	33	24.63	1.93 (0.61, 5.81)	0.296
*Yes*	14	60.87	9	39.13		
Increased needle sharing						
*No*	113	73.38	41	26.62	1.45 (0.02, 29.15)	1.000
*Yes*	2	66.67	1	33.33		
Overdosing						
*No*	108	74.48	37	25.52	2.77 (0.56, 13.71)	0.248
*Yes*	7	58.33	5	41.67		

Note: * Adjusted Odds Ratio (*aOR*) indicating the likelihood of being unable to practice COVID-19 personal protective behaviors for the ‘yes’ vs. the ‘no’ (reference group) categories for each of the substance use types.

## Data Availability

The dataset used for the current study is not publicly available because it contains information that could compromise the privacy of the research participants. The dataset is available from the corresponding author upon reasonable request.

## Abbreviations

The following abbreviations are used in this manuscript:

*aOR*	Adjusted Odds Ratio
CHC	Community Health Center
*CI*	Confidence Interval
COVID-19	Coronavirus disease 2019
GAD-7	7-item General Anxiety Disorder
HCV	Hepatitis C Virus
HERO	Hepatitis C Real Options
HIV	Human Immunodeficiency Virus
mDOT	modified Directly Observed Treatment
OTP	Opioid Treatment Program
PHQ-9	9-item Patient Health Questionnaire
PN	Patient Navigation
PWID	Persons Who Inject Drugs
SDoH	Social Drivers of Health
SVR	Sustained Virologic Response
THC	tetrahydrocannabinol
US	United States

## Appendix A

Has the COVID-19 pandemic led to any of the following (responses to each of the items below were measured on a yes/no response scale):

- You or a member of your household losing their job, having to stop working, or having to work fewer hours
- Losing childcare or having to spend more time taking care of children
- You or a member of your household losings of other sources of financial support, like food stamps
- Losing your housing or becoming homeless
- Losing your health insurance
- Difficulty paying for basic needs, including food, clothing, shelter and heat

## Appendix B

Please tell me which of the following things you have been doing in the past 2 weeks, if you would like to do these things but are not able or if you don’t see a need to do these things.

- Regularly wash my hands/use hand sanitizer
- Use disinfectants on surfaces
- Keep at least six feet of distance between you and anyone outside your immediate household
- Quarantine or self-isolate at home (defined as staying in your home with no visitors, and either no trips outside or only necessary trips outside for groceries or other essential supplies while keeping >6 feet of distance between you and anyone outside your immediate household)
- Avoid gathering in groups (of >3 people you don’t live with, e.g., to socialize, score or use drugs, to hang out), or avoid spending time in public spaces where many people are present
